# Supplementary material for: MetaQTL: a package of new computational methods for the meta-analysis of QTL mapping experiments
Source: BMC Bioinformatics. 2007 Feb 8;8:49. doi: 10.1186/1471-2105-8-49 (PMC1808479; doi:10.1186/1471-2105-8-49)
Supplement: Additional File 7 — MetaQTL Package : jar file and tutorial. This Zip archive contains both the MetaQTL JAR file and the files of the tutorial. [file 1471-2105-8-49-S7.zip › org.inra.metaqtl/doc/org/metaqtl/bio/class-use/IBioLocus.html]

Uses of Interface org.metaqtl.bio.IBioLocus


|  |  |  |
| --- | --- | --- |
| **Overview** \| **Package** \| **Class** \| **Use** \| **Tree** \| **Deprecated** \| **Index** \| **Help** | |  |
| PREV   NEXT | **FRAMES**    **NO FRAMES**     **All Classes** |  |


---


## **Uses of Interface org.metaqtl.bio.IBioLocus**

| Packages that use IBioLocus | |
| --- | --- |
| **org.metaqtl** |  |
| **org.metaqtl.adapter** |  |
| **org.metaqtl.algo** |  |
| **org.metaqtl.bio** |  |
| **org.metaqtl.bio.entity** |  |
| **org.metaqtl.bio.util** |  |
| **org.metaqtl.factory** |  |
| **org.metaqtl.main** |  |
| **org.metaqtl.util** |  |

Uses of IBioLocus in org.metaqtl

| Methods in org.metaqtl with parameters of type IBioLocus | |
| --- | --- |
| `void` | `Chromosome.attachQTL(IBioLocus[] qtls)`             Add |
| `boolean` | `ChromCluster.isDubiousLocus(IBioLocus locus)`             If a list of dubious markers has been set before then this routine checks if the given locus is not included into this list. |
| `void` | `ChromCluster.setDubiousMarker(IBioLocus[] dubious)`             This method sets the list of dubious marker. |
| `void` | `MapCluster.setDubiousMarker(IBioLocus[] dubious)`             This method sets the list of dubious marker. |

Uses of IBioLocus in org.metaqtl.adapter

| Methods in org.metaqtl.adapter that return IBioLocus | |
| --- | --- |
| `static IBioLocus` | `QtlAdapter.fromQTL(Qtl qtl)`             This method converts a `Qtl` into an object which class implements the `IBioLocus interface.` |

| Methods in org.metaqtl.adapter with parameters of type IBioLocus | |
| --- | --- |
| `static Qtl` | `QtlAdapter.toQTL(IBioLocus locus)`             This method converts an object which class implements the `IBioLocus interface into a Qtl.` |

Uses of IBioLocus in org.metaqtl.algo

Constructors in org.metaqtl.algo with parameters of type IBioLocus

`MetaMapAlgorithm(IBioGenome[] maps,
IBioGenome skeleton,
IBioLocus[] dubious,
MetaDico mrkDico)`

`QtlProjAlgorithm(IBioGenome[] maps,
IBioGenome skeleton,
IBioLocus[] dubious)`
  
          Creates a new instance of `QtlProjAlgorithm` for
the given configuration of projection.

Uses of IBioLocus in org.metaqtl.bio

| Methods in org.metaqtl.bio that return IBioLocus | |
| --- | --- |
| `IBioLocus` | `IBioAllele.getLocus()` |
| `IBioLocus` | `IBioLGroup.getLocus(java.lang.String name)` |
| `IBioLocus[]` | `IBioLGroup.loci()` |

| Methods in org.metaqtl.bio with parameters of type IBioLocus | |
| --- | --- |
| `void` | `IBioLGroup.addLocus(IBioLocus locus)` |
| `void` | `IBioLGroup.setLoci(IBioLocus[] loci)` |
| `void` | `IBioAllele.setLocus(IBioLocus locus)` |

Uses of IBioLocus in org.metaqtl.bio.entity

| Classes in org.metaqtl.bio.entity that implement IBioLocus | |
| --- | --- |
| `class` | `AlleleContainer`             Class Description Here |
| `class` | `Locus` |
| `class` | `Marker`             Class Description Here |
| `class` | `QTL`             Class Description Here |

| Fields in org.metaqtl.bio.entity declared as IBioLocus | |
| --- | --- |
| `static IBioLocus[]` | `IBioConstants.EMPTY_BIOLOCUS_ARRAY` |

| Methods in org.metaqtl.bio.entity that return IBioLocus | |
| --- | --- |
| `IBioLocus` | `LocusContainer.getLocus(java.lang.String name)` |
| `IBioLocus[]` | `LocusContainer.loci()` |

| Methods in org.metaqtl.bio.entity with parameters of type IBioLocus | |
| --- | --- |
| `void` | `LocusContainer.addLocus(IBioLocus locus)` |
| `void` | `LocusContainer.setLoci(IBioLocus[] loci)` |

Uses of IBioLocus in org.metaqtl.bio.util

| Methods in org.metaqtl.bio.util that return IBioLocus | |
| --- | --- |
| `static IBioLocus[]` | `BioLocusTypes.extractLocus(IBioLocus[] loci, int type)`             This returns extracts in the given array of loci the locus which type matches the given one. |
| `static IBioLocus[]` | `BioUtilities.sortLociByPosition(IBioLocus[] loci)`             Sort tab with loci |

| Methods in org.metaqtl.bio.util with parameters of type IBioLocus | |
| --- | --- |
| `static IBioLocus[]` | `BioLocusTypes.extractLocus(IBioLocus[] loci, int type)`             This returns extracts in the given array of loci the locus which type matches the given one. |
| `static IBioLocus[]` | `BioUtilities.sortLociByPosition(IBioLocus[] loci)`             Sort tab with loci |

Uses of IBioLocus in org.metaqtl.factory

| Methods in org.metaqtl.factory that return IBioLocus | |
| --- | --- |
| `static IBioLocus[]` | `DubiousLocusFactory.read(java.io.Reader reader)`             This routine reads the list of dubious markers from the given file location and returns if succeed an array of `IBioLocus` objects. |

Uses of IBioLocus in org.metaqtl.main

| Methods in org.metaqtl.main that return IBioLocus | |
| --- | --- |
| `protected static IBioLocus[]` | `MetaMain.getDubious(java.lang.String dubFile)` |

Uses of IBioLocus in org.metaqtl.util

| Methods in org.metaqtl.util with parameters of type IBioLocus | |
| --- | --- |
| `static int` | `LocusProperties.getMetaOccurence(IBioLocus locus)` |
| `static boolean` | `LocusProperties.isDubiousOrderLocus(IBioLocus locus)`             Returns true if the given locus has a property order.dubious set to one, false otherwise |

---


|  |  |  |
| --- | --- | --- |
| **Overview** \| **Package** \| **Class** \| **Use** \| **Tree** \| **Deprecated** \| **Index** \| **Help** | |  |
| PREV   NEXT | **FRAMES**    **NO FRAMES**     **All Classes** |  |


---
